# The Effects of Aβ_1-42_ Binding to the SARS-CoV-2 Spike Protein S1 Subunit and Angiotensin-Converting Enzyme 2

**DOI:** 10.3390/ijms22158226

**Published:** 2021-07-30

**Authors:** John Tsu-An Hsu, Chih-Feng Tien, Guann-Yi Yu, Santai Shen, Yi-Hsuan Lee, Pei-Chien Hsu, Yun Wang, Po-Kuan Chao, Huey-Jen Tsay, Feng-Shiun Shie

**Affiliations:** 1 Institute of Biotechnology and Pharmaceutical Research, National Health Research Institutes, Miaoli County 35053, Taiwan; tsuanhsu@nhri.edu.tw; 2 National Institute of Infectious Diseases and Vaccinology, National Health Research Institutes, Miaoli County 35053, Taiwan; chemltt2010@gmail.com (C.-F.T.); guannyiy@nhri.edu.tw (G.-Y.Y.); 3 Antaimmu BioMed Co., Ltd., Hsinchu 30078, Taiwan; Santai.Shen@antaimmu.com; 4 Department and Institute of Physiology, National Yang-Ming University, Taipei 11221, Taiwan; yhlee3@nycu.edu.tw (Y.-H.L.); pachann99@gmail.com (P.-C.H.); 5 Department and Institute of Physiology, National Yang Ming Chiao Tung University, Taipei 11221, Taiwan; 6 Center for Neuropsychiatric Research, National Health Research Institutes, Miaoli County 35053, Taiwan; ywang@nhri.edu.tw (Y.W.); hubert@nhri.edu.tw (P.-K.C.); 7 Institute of Neuroscience, School of Life Science, National Yang-Ming University, Taipei 11221, Taiwan; hjtsay@ym.edu.tw

**Keywords:** Alzheimer’s disease, Aβ, COVID-19, SARS-CoV-2 spike protein, ACE2

## Abstract

Increasing evidence suggests that elderly people with dementia are vulnerable to the development of severe coronavirus disease 2019 (COVID-19). In Alzheimer’s disease (AD), the major form of dementia, β-amyloid (Aβ) levels in the blood are increased; however, the impact of elevated Aβ levels on the progression of COVID-19 remains largely unknown. Here, our findings demonstrate that Aβ_1-42_, but not Aβ_1-40_, bound to various viral proteins with a preferentially high affinity for the spike protein S1 subunit (S1) of severe acute respiratory syndrome coronavirus 2 (SARS-CoV-2) and the viral receptor, angiotensin-converting enzyme 2 (ACE2). These bindings were mainly through the C-terminal residues of Aβ_1-42_. Furthermore, Aβ_1-42_ strengthened the binding of the S1 of SARS-CoV-2 to ACE2 and increased the viral entry and production of IL-6 in a SARS-CoV-2 pseudovirus infection model. Intriguingly, data from a surrogate mouse model with intravenous inoculation of Aβ_1-42_ show that the clearance of Aβ_1-42_ in the blood was dampened in the presence of the extracellular domain of the spike protein trimers of SARS-CoV-2, whose effects can be prevented by a novel anti-Aβ antibody. In conclusion, these findings suggest that the binding of Aβ_1-42_ to the S1 of SARS-CoV-2 and ACE2 may have a negative impact on the course and severity of SARS-CoV-2 infection. Further investigations are warranted to elucidate the underlying mechanisms and examine whether reducing the level of Aβ_1-42_ in the blood is beneficial to the fight against COVID-19 and AD.

## 1. Introduction

Coronavirus disease 2019 (COVID-19) is caused by infection with severe acute respiratory syndrome coronavirus 2 (SARS-CoV-2), which is the recently identified coronavirus with positive single-strand RNA [1]. Most individuals infected with this virus are asymptomatic or suffer from mild illness. However, nearly 20% of patients, especially older individuals and those with underlying medical conditions, develop severe lung disease, which may lead to multi-organ failure and death. While the overall estimated global fatality rate of COVID-19-positive individuals is approximately 1–2%, this fatality rate varies considerably with age [2]. Although the mechanisms underlying the development of severe COVID-19 are unclear, many studies have indicated that infected individuals who are obese and/or have chronic diseases, such as diabetes mellitus, hypertension, or cancer, tend to be hospitalized with severe clinical manifestations of COVID-19 [3]. There is also increasing evidence that patients with severe COVID-19 manifest neurological symptoms [4,5,6,7], though viral RNA is rarely found in the brain. Importantly, some studies have shown that people with dementia are more vulnerable to severe COVID-19 [8,9,10,11], although the higher incidence of COVID-19 in nursing homes has been attributed to the lack of ability of dementia patients to comply with hygiene standards. However, it is unclear whether the pathology of patients with dementia directly affects the development of severe COVID-19.

Alzheimer’s disease (AD) accounts for nearly 80% of dementia cases, but no reports on the association between AD and the severity of COVID-19 have been published. Since β-amyloid (Aβ) is the major pathological component of senile plaques in the brains of AD patients [12,13], it has been a promising target used by many pharmaceutical companies for immuno-therapeutic treatment [14]. Among the Aβ species mostly comprising Aβ_1-40_ and Aβ_1-42_, derived from amyloid precursor protein (APP) via secretase activities, Aβ_1-42_ has been shown to be more toxic to neurons than Aβ_1-40_ and is a stronger indicator of AD [15]. Many mutations around Aβ sequences result in early onset or familial AD via increased Aβ expression or accumulation. Aβ mutations also affect the physicochemical properties of residues far from their mutation sites and are prone to self-aggregation [16]. Previous studies have reported anti-microbial activity of Aβ, and infection with the herpes simplex virus has been proposed to be involved in Aβ plaque formation [17,18,19]. Moreover, certain underlying conditions, such as obesity and diabetes mellitus, that increase vulnerability to severe COVID-19, also increase circulating Aβ levels and affect the progression of AD [20,21]. Therefore, the effects of Aβ on SARS-CoV-2 infection were explored in this study.

For this purpose, we evaluated the binding of Aβ_1-42_ to the receptor of SARS-CoV-2, angiotensin-converting enzyme 2 (ACE2), and various viral proteins, including the spike protein S1 subunit (S1) of SARS-CoV-2, influenza A (H1N1), and Middle East respiratory syndrome coronavirus (MERS-CoV). Viral infection with the SARS-CoV-2 pseudovirus was examined in the presence or absence of Aβ_1-42_, and associated cytokine expression was examined in A549 cells, an immortalized human alveolar epithelial cell line. Since previous reports have shown that the clearance of Aβ_1-42_ in the circulation was reduced in AD models [22], a surrogate mouse model with intravenous inoculation of Aβ_1-42_ was used to investigate whether the extracellular domain of the spike protein (SP_ECD_) trimers of SARS-CoV-2 in the blood affect Aβ_1-42_ homeostasis. Thus, this study explored the nature of interactions between Aβ_1-42_ and the S1 of SARS-CoV-2 to assess the possible impacts of Aβ_1-42_ during the progression of COVID-19.

## 2. Results

### 2.1. Aβ_1-42_ Binds to Viral Proteins with a High Affinity to the S1 of SARS-CoV-2

**Figure 1:**
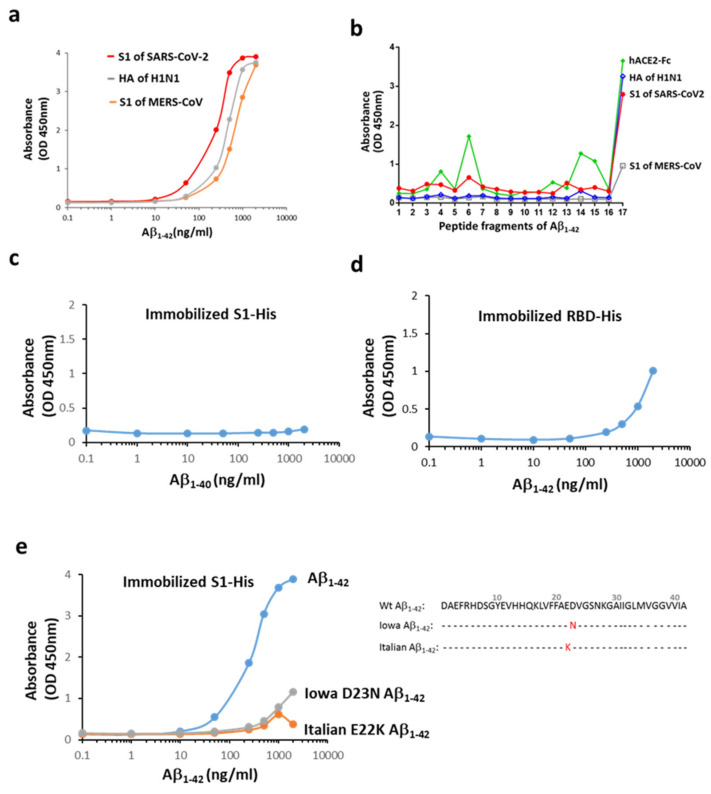
Interactions between Aβ_1-42_ and the S1 of SARS-CoV-2. (**a**) Aβ_1-42_ differentially binds to the immobilized surface proteins of three viruses responsible for recent pandemics. The order of potency of binding based on the estimated EC_50_ was the S1 of SARS-CoV-2 (200–325 ng/mL) > HA of H1N1 (HA, 372–507 ng/mL) > S1 of MERS-CoV (599–860 ng/mL). (**b**) Linear epitope mapping was performed using 17 fragment peptides of Aβ_1-42_ (details in Supplementary Figure S2), and the binding ability is indicated by OD. Aβ_1-42_ interacted with the S1 of SARS-CoV-2, mainly in the last probe containing the hydrophobic Aβ residues of 33–42. A similar binding pattern on Aβ_1-42_ was observed for HA of H1N1, and to a lesser extent, for the S1 of MERS-CoV. hACE2-Fc presents three more binding sites (OD > 0.8) across the entire sequence of Aβ_1-42_ in addition to a major binding site on the C-terminal end. The following binding potency was compared by OD. (**c**) Aβ_1-40_ did not bind to the S1 of SARS-CoV-2. (**d**) Aβ_1-42_ bound weakly to the immobilized His-tagged RBD of SARS-CoV-2 as compared to the S1 protein. (**e**) Iowa D23N and Italian E22K Aβ_1-42_ mutants exerted marked reduction in the S1 binding, and amino acid sequences for normal human (Wt) and mutated Aβ_1-42_ are presented. Data are presented as mean values from two independent experiments.

To examine the interaction between Aβ_1-42_ and the surface proteins of three families of viruses responsible for recent pandemics (i.e., SARS-CoV-2, H1N1, and MERS-CoV), a functional ELISA assay was conducted. As shown in Figure 1a, Aβ_1-42_ binds to all the viral proteins with a preferentially high affinity for S1 of SARS-CoV-2 (binding with S1 of SARS-CoV-2 > hemagglutinin of H1N1 > S1 of MERS-CoV). The binding propensity using linear epitope mapping further revealed that the S1 of SARS-CoV-2 bound selectively to Aβ_1-42_ at the last C-terminal fragment (33–42 amino acids), but not to fragment 16 containing residues 31-40 (Figure 1b). In line with these observations, the S1 of SARS-CoV-2 did not interact with Aβ_1-40_ (Figure 1c). These data suggested that the S1 of SARS-CoV-2 interacted with Aβ_1-42_ in the C-terminus hydrophobic core region, and the last two amino acids (41–42) were critical for the binding. Our data also show that the binding of Aβ_1-42_ to the receptor-binding domain (RBD) of SARS-CoV-2 was weaker compared to that observed with the S1 of SARS-CoV-2, suggesting that the main binding site of Aβ_1-42_ was not located at the RBD of S1 (Figure 1d). Additionally, two Aβ_1-42_ mutants (Iowa D23N and Italian E22K of familial AD), where the mutation in the middle of Aβ_1-42_ sequence causes a conformational change in the C-terminus, exhibited very low binding to the immobilized S1 of SARS-CoV-2 (Figure 1e). These data suggest that the interaction of Aβ_1-42_ and S1 of SARS-CoV-2 is determined by its C-terminal conformation.

### 2.2. Aβ_1-42_ Binds to hACE2 and Strengthens the Binding of the S1 of SARS-CoV-2 to hACE2

**Figure 2:**
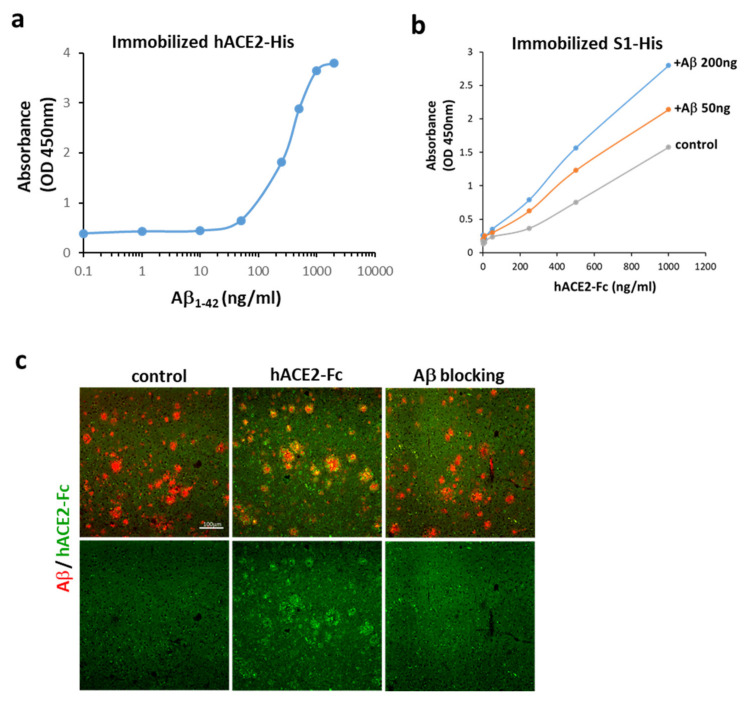
Interactions between Aβ_1-42_ and ACE2. (**a**) Aβ_1-42_ bound to immobilized hACE2-His (the estimated EC_50_ is 274–363 ng/mL), suggesting an apparent interaction between Aβ_1-42_ and hACE2. (**b**) ACE2-Fc bound to immobilized S1 of SARS-CoV-2 in the absence of Aβ_1-42_ (control), and this binding was increased by pre-incubation of immobilized S1 of SARS-CoV-2 with Aβ_1-42_ at 50 and 200 ng, whose binding potency was compared by OD. (**c**) As shown by confocal microscopy, exogenous hACE2-Fc (green) co-localized with Aβ plaques (red) in the brain tissue sections of APP/PS1 mice (vehicle shown in left panels vs. hACE-Fc in middle panels). Pre-incubation of hACE-Fc with Aβ_1-42_ (right panel) completely blocked hACE2-Fc/Aβ plaque interaction (as indicated by Aβ blocking). Scale bar: 100 μm. Data of the binding assay are presented as mean values from two independent experiments.

As shown in Figure 1b, human ACE2 conjugated with Fc (hACE2-Fc) interacted with Aβ_1-42_ via multiple sites in addition to the C-terminus, as seen in linear epitope mapping of fragments #4, 6, 14, and 15 of Aβ_1-42_. Similarly, an apparent interaction between Aβ_1-42_ and hACE2 was also found using immobilized hACE2-His (Figure 2a). To investigate the effects of Aβ_1-42_ on the binding between the S1 of SARS-CoV-2 and ACE2, a competitive ELISA binding assay was applied, and Aβ_1-42_ was pre-incubated with immobilized S1 of SARS-CoV-2 followed by the addition of hACE2-Fc. As shown in Figure 2b, hACE2-Fc bound to immobilized S1 of SARS-CoV-2 in the absence of Aβ_1-42_, and this binding was increased by pre-incubation of immobilized S1 of SARS-CoV-2 with Aβ_1-42_ in a dose-dependent manner. Based on the aforementioned results, it can be explained that the RBD on the S1 of SARS-CoV-2 is not occupied by Aβ_1-42_, and is accessible to hACE2-Fc. This condition also allows the interaction between hACE2-Fc and Aβ_1-42_ via the multiple interacting sites, leading to a stronger binding between the S1 of SARS-CoV-2 and hACE2-Fc. These data suggest that Aβ_1-42_ binds to hACE2 and strengthens the binding of the S1 of SARS-CoV-2 to hACE2. Additionally, the interaction between the pathological Aβ peptides in AD and hACE2 was examined by using an AD mouse model expressing human Aβ peptides. The brain sections of APP/PS1 mice loaded with Aβ deposition were exogenously applied with hACE2-Fc, and data show that hACE2-Fc co-localized with Aβ plaques, as observed in the confocal images (Figure 2c). Not surprisingly, pre-incubation of hACE2-Fc with Aβ_1-42_ completely blocked this hACE2-Fc/Aβ plaque interaction. These findings support our speculation that human Aβ peptides in an AD mouse model bind to hACE2.

### 2.3. Aβ_1-42_ Increases the Infectivity of SARS-CoV-2 Pseudovirus and IL-6 in Host Cells

**Figure 3:**
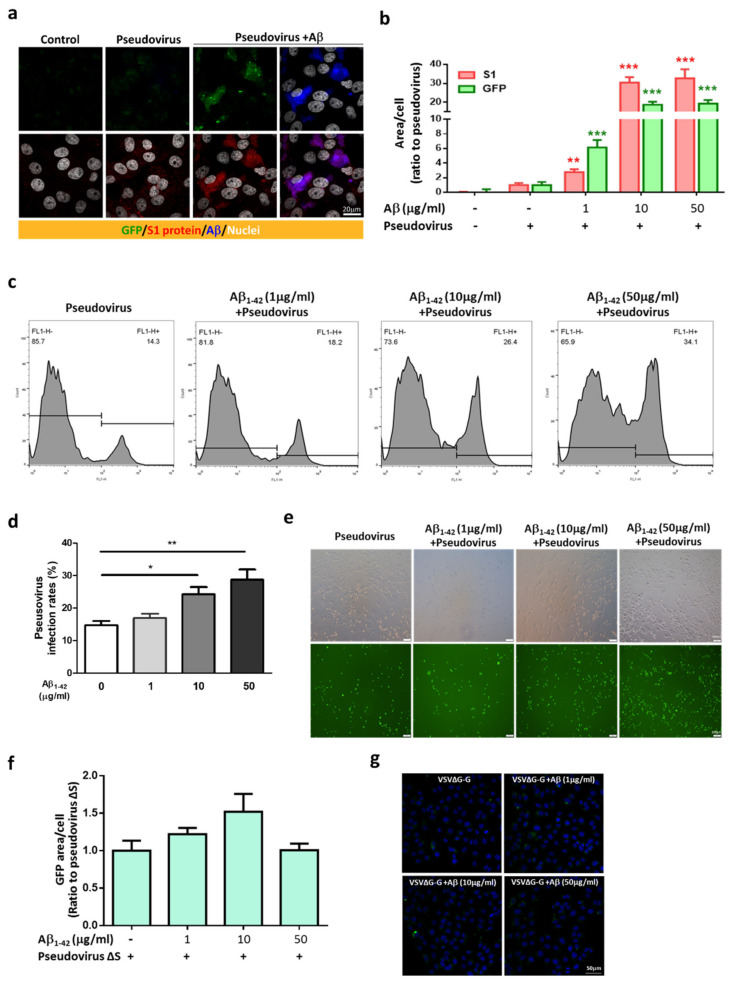
Aβ_1-42_ increases the infectivity of SARS-CoV-2 pseudovirus. (**a**) GFP fluorescence of SARS-CoV-2 (green, upper panels) and immuno-reactivity of the S1 of SARS-CoV-2 (red, lower panels) were barely detected 2 h post-infection in Vero E6 cells with vehicle (control) or SARS-CoV-2 pseudovirus alone (pseudovirus), which were robustly elevated in the infected cells with Aβ_1-42_ treatment (pseudovirus+Aβ). Intriguingly, Aβ_1-42_ (blue) was co-localized with the immuno-reactivity of the S1 of SARS-CoV-2 (shown as purple in right lower image). Scale bar: 20 μm. (**b**) Quantification of GFP fluorescence and the S1 immuno-reactivity in Vero E6 cells with viral infection and Aβ_1-42_ treatments is shown. Aβ_1-42_ (1 µg/mL) significantly increased GFP expression (6.15 ± 1.02-fold increase compared to the control, *p* < 0.001) and S1 immuno-reactivity (2.77 ± 0.41-fold increase compared to control, *p* < 0.01). Further increases in GFP expression and S1 immuno-reactivity were found at 10 µg/mL (18.71 ± 1.58-fold increase compared to the control, *p* < 0.001, and 30.41 ± 2.91-fold increase compared to control, *p* < 0.001, respectively) and 50 µg/mL of Aβ_1-42_ (19.3 ± 1.88-fold increase compared to the control, *p* < 0.001, and 32.69 ± 4.79-fold increase compared to the control, *p* < 0.001, respectively). (**c**) Representative histograms of flow cytometry indicated that Aβ_1-42_ (1 to 50 µg/mL) increased GFP fluorescence (% total counts) in cells. (**d**) Quantification of the viral infectivity from flow cytometry is presented as infection rates (%, for details, please see Materials and Methods). The infection rates for cells treated with Aβ_1-42_ at doses of 1, 10, and 50 µg/mL were 16.87% ± 1.32% (ns), 24.22% ± 2.24% (* *p* < 0.05), and 28.7% ± 3.2% (** *p* < 0.01), respectively, compared to the controls (14.65% ± 1.32%; *n* = 6 per group). (**e**) Representative photomicrographs of phase contrast (upper panels) and fluorescent images (lower panels) are presented. Scale bars: 100 μm. (**f**) Viral infectivity of pseudovirus VSVΔG-G was measured by total GFP fluorescence with DAPI by confocal microscopy in Vero E6 cells. Treatment with Aβ_1-42_ (0, 1, 10, 50 µg/mL) did not significantly increase viral infection of pseudovirus VSVΔG-G in Vero E6 cells after a 2 h incubation (*p* = 0.0571). (**g**) Representative confocal images indicated that the expression of GFP was minimal after co-treatment with pseudovirus VSVΔG-G and Aβ_1-42_. Scale bar: 50 μm.

**Figure 4:**
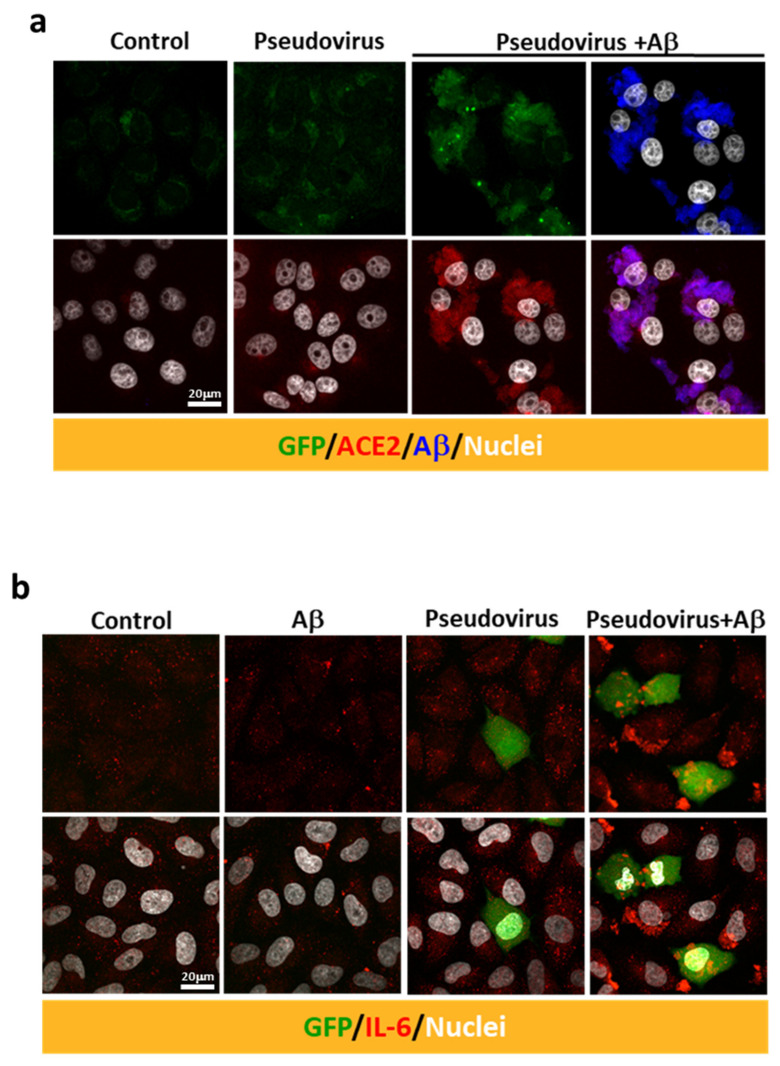
Aβ_1-42_ increases the intracellular immuno-reactivity of IL-6 in a SARS-CoV-2 pseudovirus infection model. (**a**) Immuno-reactivity of endogenous ACE2 (in red) was detected at relatively low levels in the controls. Treatment with Aβ_1-42_ (10 µg/mL) and SARS-CoV-2 pseudovirus increased the expression of ACE2 (red, lower panel) as well as the co-localization of ACE2 and Aβ_1-42_ (in blue) in Vero E6 cells. (**b**) Intracellular IL-6 expression was evaluated by confocal microscopy after infection with SARS-CoV-2 pseudovirus in the presence or the absence of Aβ_1-42_ (50 µg/mL) in human A549 alveolar epithelial cells. GFP (in green) was abundantly expressed in a few cells 17 h post-infection. Minimal intracellular IL-6 immuno-reactivity was observed in cells receiving either Aβ_1-42_ or SARS-CoV-2 pseudovirus alone. In contrast, infection with SARS-CoV-2 pseudovirus in the presence of Aβ_1-42_ increased intracellular IL-6 immuno-reactivity (in red). Scale bars: 20 μm.

An in vitro model was used to further explore the effects of Aβ_1-42_ on viral infection in host cells, and the SARS-CoV-2 pseudovirus expressing the full-length spike protein of SARS-CoV-2 on the viral surface and green fluorescent protein (GFP) and Vero E6 cells were applied. Co-treatment with Aβ_1-42_ increased the expression of GFP and the S1 immuno-reactivity in cells 2 h post-infection, and Aβ_1-42_ was co-localized with S1 immuno-reactivity as observed with confocal microscopy (Figure 3a). Since the spike protein of SARS-CoV-2 pseudovirus is not designed to be amplified after cell infection, an increased level of S1 immuno-reactivity implies a higher infection rate. In contrast, relatively low levels of S1 and GFP were detected in the cells infected with the SARS-CoV-2 pseudovirus in the absence of Aβ_1-42_, suggesting an important role played by Aβ_1-42_ in viral infection. Aβ_1-42_ significantly increased SARS-CoV-2 pseudovirus infection in a dose-dependent manner, as demonstrated by semi-quantification of GFP fluorescence and S1 immuno-reactivity in Vero E6 cells (Figure 3b). A similar effect was also demonstrated by flow cytometry 24 h post-infection (Figure 3c), in which Aβ_1-42_ increased SARS-CoV-2 pseudovirus infection rates in a dose-dependent manner in Vero E6 cells (Figure 3d). Representative photomicrographs of phase contrast (upper panel) and fluorescent images (lower panel) are presented in Figure 3e. In contrast, Aβ_1-42_ showed a less remarkable effect on the infectivity of a control pseudotyped virus without the S protein of SARS-CoV-2 (VSVΔG-G) in Vero E6 cells (Figure 3f,g), suggesting that S protein was involved in Aβ_1-42_-enhanced SARS-CoV-2 pseudovirus infection. Furthermore, Aβ_1-42_ co-localized with endogenous ACE2 in Vero E6 cells, as demonstrated by confocal microscopy (Figure 4a). These data support the notion that Aβ_1-42_ increases viral infectivity through interaction with the viral S1 protein and the ACE2 protein in host cells. Since IL-6 levels are important indicators for the severity of COVID-19, we examined Aβ_1-42_-mediated changes in IL-6 production in A549 cells, an immortalized human alveolar epithelial cell line. Data show that the IL-6 immuno-reactivity per cell at 17 h post-infection was not significantly altered by SARS-CoV-2 pseudovirus infection (11.92 ± 1.23 a.u.) or by treatment with Aβ_1-42_ alone (6.12 ± 1.11 a.u.) compared to the control (8.56 ± 0.66 a.u.). In contrast, intracellular IL-6 immuno-reactivity was significantly increased by treatment with the SARS-CoV-2 pseudovirus in the presence of Aβ_1-42_ (29.01 ± 3.94 a.u., *p* < 0.001) compared to SARS-CoV-2 pseudovirus alone, and representative images are illustrated in Figure 4b. These data suggest that Aβ_1-42_ increases not only the infectivity of SARS-CoV-2 pseudovirus but also inflammation in host cells.

### 2.4. The Extracellular Domain (ECD) of the S Protein of SARS-CoV-2 (SP_ECD_) Reduces Aβ Clearance in the Blood

**Figure 5:**
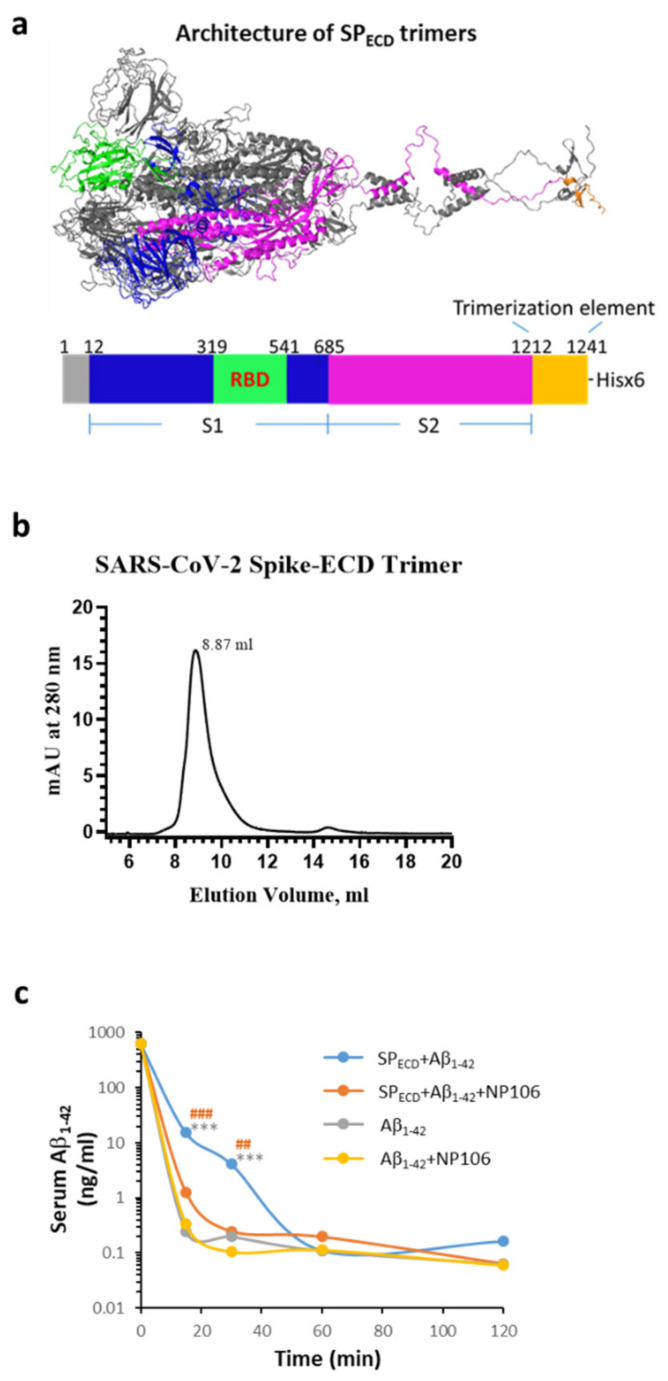
Effects of the extracellular domain (ECD) of the S protein of SARS-CoV-2 (SP_ECD_) trimers on Aβ_1-42_ clearance in the blood. (**a**) The architecture of SP_ECD_ trimers, including the spike protein and trimerization element, produced by PyMOL program using the FUSE command (for details, see Materials and Methods). SP_ECD_ trimers were constructed using a 27-amino acid trimerization domain (foldon) and 3 amino acids for the restriction enzyme site fused in the C-terminal of SP_ECD_ (1212-1241). The monomer domain organization of SP_ECD_ containing the S1, the S2, a trimerization element, and His-tag (x6) is indicated, which resulted in 18 His in an SP_ECD_ trimer. (**b**) His-tagged SP_ECD_ trimers were subjected to size exclusion chromatography, and a major peak (approximately 96.66%, eluted at 8.87 mL) corresponding to the trimeric structure was estimated to be approximately 814 kDa. (**c**) A surrogate mouse model for the evaluation of Aβ clearance during the viral infection was established by intravenous injection of Aβ_1-42_ in the presence or absence of SP_ECD_ trimers using wild-type C57/BL6 mice. Serum Aβ_1-42_ was measured at 15 min, 30 min, 60 min, and 120 min after injection. Mice that received co-injection of SP_ECD_ trimers with Aβ_1-42_ (blue line, *n* = 5) exhibited a slower clearance curve of serum Aβ_1-42_, especially 15 and 30 min after injection, than those injected with Aβ_1-42_ alone (gray line, *n* = 5). Co-injection of NP106 with SP_ECD_ trimers and Aβ_1-42_ (orange line, *n* = 4) significantly prevented such slowing effects, while the clearance curve of serum Aβ_1-42_ was not affected by NP106 (yellow line, *n* = 5). Data are presented as mean values of serum Aβ_1-42_ levels at the specified time point from animals used in this study *** *p* < 0.001, SP_ECD_ + Aβ_1-42_ compared to Aβ_1-42_; ## *p* < 0.01, ### *p* < 0.001, SP_ECD_ +Aβ_1-42_ compared to SP_ECD_ + Aβ_1-42_+ NP106.

We next investigated whether viral protein, in turn, affected the clearance of Aβ_1-42_. Since circulating Aβ levels increase in AD and other disorders (i.e., obesity and diabetes mellitus, conditions that have been reported to increase vulnerability to severe COVID-19), serum Aβ_1-42_ was examined at indicated time points in a surrogate mouse model with intravenous inoculation of Aβ_1-42_. The SP_ECD_ of SARS-CoV-2 in a trimer structure was constructed and was used to mimic viral infection (Figure 5a,b depict features of SP_ECD_ trimers). Aβ_1-42_ was intravenously injected into C57/BL6 mice with or without SP_ECD_ trimers. Data show that co-treatment with SP_ECD_ trimers significantly reduced the clearance of serum Aβ_1-42_, especially at 15 and 30 min after injection (Figure 5c), which was prevented by the co-administration of NP106, a novel antibody specific for human Aβ (Supplementary Figure S1). However, the clearance curve of serum Aβ_1-42_ was not affected by NP106. These results imply that the clearance of Aβ_1-42_ can be dampened during SARS-CoV-2 infection. It is feasible to speculate that these effects may be due to the aforementioned interactions between Aβ_1-42_ and the S1 of SARS-CoV-2.

## 3. Discussion

Although the anti-microbial activity of Aβ and the possible role of herpes simplex virus infection in Aβ plaque formation have been reported, the effects of Aβ on the progression of COVID-19 remain unknown. To the best of our knowledge, this is the first study to report high-affinity binding of Aβ_1-42_ to ACE2 and S1 of SARS-CoV-2 and two surface proteins on other viral families. Importantly, our data suggest mechanisms by which Aβ_1-42_ may enhance SARS-CoV-2 infection/inflammation, and show that viral infection reciprocally affects Aβ_1-42_ clearance. These findings highlight the pivotal role of Aβ_1-42_ in increasing SARS-CoV-2 intrusions and suggest caution towards the potential impact of COVID-19 in patients with pre-existing Aβ abnormalities, such as those with AD or metabolic disorders. Previous reports have indicated that clearance of Aβ_1-42_ in the circulation is reduced in AD. Although SARS-CoV-2 was rarely detected in the brain, the increase in the S protein in the circulation after SARS-CoV-2 infection may lead to aberrant Aβ homeostasis and promote neurological dysfunction. In fact, the interactions between Aβ peptide and other proteins may have positive or negative impacts on the progression of AD. For example, transthyretin tetramer binding to Aβ prevents Aβ aggregation and/or promotes Aβ clearance [23,24], and the binding of calmodulin to Aβ has been reported to modulate neuronal functioning [25]. In contrast, the binding of tau to Aβ accelerates Aβ fibrillogenesis and tau phosphorylation [26]. Aβ peptide also binds to trace metals such as copper, zinc, iron, aluminum, and manganese, and aberrant homeostasis of these trace metals is associated with AD [27]. The close interactions among Aβ_1-42_, S1 of SARS-CoV-2, and ACE2 are likely to be have a negative impact on both AD and COVID-19. However, the underlying mechanisms should be explored further.

Notably, the expression of ACE2 has been reported to be reduced in some AD patients [28], and increasing ACE2 to promote anti-inflammatory functions has been hypothesized to be beneficial for AD [29]. Therefore, individuals with reduced expression levels of ACE2 may be protected from viral infection. However, contradictory findings show that ACE2 was upregulated in the hippocampus of AD patients, albeit not correlated with the severity of AD [30]. Therefore, Aβ pathology might be an important contributor to the possible association between AD and severe COVID-19. Further investigations are urgently warranted to examine whether a pre-existing Aβ abnormality in circulation is critical for viral infection and for induction of cytokine storms in patients with severe COVID-19.

In this study, we observed that Aβ_1-40_ and two Aβ_1-42_ mutants exhibited markedly low binding to the immobilized S1 of SARS-CoV-2. In addition, Aβ_1-42_ bound weakly to RBD of SARS-CoV-2 and did not prevent the binding of ACE2 to the S1 of SARS-CoV-2, although a recent study predicts the interaction between Aβ and RBD of SARS-CoV-2 by a docking model [31]. Our findings suggest that the C-terminal conformation of Aβ_1-42_ is critical for the interaction with S1 of SARS-CoV-2, and that blockade of the C-terminal end of Aβ_1-42_ and/or removal of Aβ_1-42_ by a specific antibody may suppress its effects on the viral infection. The ability of Aβ_1-42_ to bind to various viral proteins suggests that other viral infections may also share similar mechanisms of interactions with Aβ pathology, which could be an attractive therapeutic target for current and future viral diseases. Therefore, it would be valuable to research anti-Aβ immuno-therapy as a potential solution to ameliorate the course and severity of COVID-19 and other diseases caused by coronaviruses.

In conclusion, our findings suggest that Aβ_1-42_ may play an important role in the development of severe COVID-19. The effects of Aβ pathology on the viral infection of SARS-CoV-2 and the induction of IL-6 in host cells involve close interactions between Aβ1-42 and the S1 of SARS-CoV-2 and ACE2. The underlying mechanisms and the effects of enhancing Aβ clearance in the blood to fight the deterioration of COVID-19 merit further investigation.

## 4. Materials and Methods

### 4.1. Animals

APP/PS1 transgenic mice (No. 005864) were purchased from the Jackson Laboratory and wild-type mice (C57/BL6) were purchased from the National Laboratory Animal Center (Taiwan, Taipei). Animals were housed under conditions of controlled room temperature (24 °C ± 1 °C) and humidity (55–65%) with a 12:12 h (0700–1900 h) light–dark cycle. Experiments were performed using APP/PS1 transgenic mice and the wild-type mice as approved by the Institutional Animal Care and Use Committees of National Health Research Institutes (NHRI, Protocol No: NHRI-IACUC-108153-M1).

### 4.2. Generation of SARS-CoV-2 Pseudovirus

The protocol for the generation of pseudovirus VSVΔG-GFP/G (VSVΔG-G) was adapted from the literature [32] with slight modifications. Briefly, BHK-21 cells (kindly provided by Dr. Andrew Yueh, NHRI, Taiwan) were maintained in minimum essential medium (MEM) alpha medium (Hyclone from Thermo Fisher, Waltham, MA, USA) containing 10% fetal bovine serum (FBS), 1× penicillin streptomycin solution (Corning, Glendale, AZ, USA), and 1× 4-(2-hydroxyethyl)-1-piperazineethanesulfonic acid (HEPES) buffer (Biological Industries, Cromwell, CT, USA) at 37 °C in a 5% CO_2_ incubator. Cells in 6-well plates were infected with a vaccinia T7 virus at a multiplicity of four. After 45 min, the BHK-21 cells were transfected with a mixture containing a total of 5 µg rVSV-ΔG-GFP-2.6 (Kerafast# EH1026, Boston, MA, USA), 8 µg pBS-G-ΦT (Kerafast# EH1016), 1 µg pBS-L-ΦT (Kerafast# EH1015), 3 µg pBS-N-ΦT (Kerafast# EH1013), and 5 µg pBS-P-ΦT (Kerafast# EH1014) using Lipofectamine 2000 (Thermo Fisher, Waltham, MA, USA) according to the manufacturer’s instructions. Six hours after transfection, the medium was removed and replaced with 5% FBS in MEM alpha medium. The virus supernatants were collected 48 h post-transfection, filtered with a 0.22 µm filter, and stored at −80 °C. For amplification of the recovered virus, the BHK-21 cells were transfected with 2 µg VSV-G plasmid in 6-well plates using Lipofectamine 2000. The next day, BHK-21 cells were infected with clarified virus supernatants. The supernatants were centrifuged 48 h post-transfection at 450× *g* for 10 min, subsequently filtered, and aliquots were stored at −80 °C. The titer of VSVΔG-G pseudovirus was determined by a median tissue culture infectious dose TCID50 assay. To generate VSVΔG-GFP/SARS-CoV-2 spike pseudotyped virus (SARS-CoV-2 pseudovirus), the BHK-21 cells were transfected with pVax1-nCoV full-length spike (from strain Wuhan-Hu-1, MN908947.3) using Lipofectamine 2000. The next day, the transfected cells were infected with VSVΔG-GFP/G at a multiplicity of five. Two hours later, the medium was removed and replaced with 2% FBS in MEM alpha medium. After 24 h, the clarified supernatant was centrifuged at 1320× *g* for 10 min and aliquots were stored at −80 °C.

### 4.3. Confocal Microscopy and Flow Cytometry for Measuring the Pseudoviral Infection in Vero E6 Cells

Vero E6 cells (kindly provided by Dr. Shiow Ju Lee, NHRI, Taiwan) were regularly maintained in DMEM/high-glucose (Hyclone) culture medium containing 10% FBS and 1× PS solution (Corning) at 37 °C in a 5% CO_2_ incubator. The Vero E6 cells (2.5 × 104 cells) were seeded overnight in a 4-well chambered slide for immuno-fluorescence microscopy or in a 24-well plate for flow cytometry. Before infection, Aβ_1-42_ (GeneZyme Biotechnology, Miaoli County, Taiwan) at 1, 10, and 50 µg/mL was separately added into cell culture in a final volume of 300 µL, along with 3.25 × 103 focus forming units of the SARS-CoV-2 pseudovirus, and incubated at 37 °C for 1 h. Culture medium was then replaced with the pseudovirus–Aβ_1-42_ mixture for infection. After 2 h of infection, medium was removed and cells were cultivated in fresh MEM alpha culture medium containing 2% FBS for an additional 2 h for immuno-fluorescent microscopy or 24 h for flow cytometry. For immuno-fluorescence microscopy, the cells were fixed with 4% paraformaldehyde in 1× PBS followed by storage in 1× PBS at 4 °C until use. Vero E6 cells were incubated with three SARS-CoV-2 S1-specific antibodies (human IgG) that have different binding epitopes (ECD37, ECD45, and ECD49, kindly provided by Antaimmu BioMed) at a 1:200 dilution (5 µg/mL) and with NP106 (2 µg/mL) in 1× PBS with 0.1% Tween 20 for 4 h at room temperature to detect the S1 protein of SARS-CoV-2 pseudovirus and Aβ_1-42_, respectively. Following 2 h of incubation with DyLight 650-conjugated donkey anti-human IgG and Alexa Fluo 594-conjugated donkey anti-mouse IgG (Thermo Fisher) at a 1:200 dilution in 1× PBS with 0.1% Tween 20, chambered slides were covered with mounting medium (Vector lab, Burlingame, CA, USA) containing 4,6-diamidino-2-phenylindole (DAPI) to label nuclei and were subjected to confocal microscopy. Images of S1 protein of SARS-CoV-2 pseudovirus (labeled with DyLight 650-conjugated donkey anti-human IgG) were presented in red pseudocolor, while those of Aβ_1-42_ (labeled with Alexa Fluo 594-conjugated donkey anti-mouse IgG) were presented in blue pseudocolor. A similar immunostaining protocol was also used for ACE2 (rabbit IgG, Novus Biologicals, Littleton, CO, USA) in Vero E6 cells. Alexa Fluo 594-conjugated donkey anti-rabbit IgG (Thermo Fisher) was used as a secondary antibody and the images were presented in red pseudocolor. Aβ_1-42_ was labeled by NP106 (2 µg/mL) followed by Alexa Fluo 647-conjugated donkey anti-mouse IgG (Thermo Fisher) and the images were presented in blue pseudocolor. Fluorescent intensity of cellular GFP was assessed using excitation by laser set at Alexa Fluo 488 nm. Semi-quantification of the SARS-CoV-2 pseudovirus infectivity was measured by total fluorescent area per DAPI-positive cell, obtained after assessment of GFP fluorescence and S protein immuno-reactivity. Randomly selected cells from the non-infected group (control, including 1198 cells), SARS-CoV-2 pseudovirus-infected group (including 1761 cells), and SARS-CoV-2 pseudovirus-infected plus Aβ_1-42_ groups at 1 (including 1061 cells), 10 (including 1769 cells), or 50 µg/mL (including 1753 cells) were used in three-chambered slides, and data are presented as ratios with respect to the SARS-CoV-2 pseudovirus-infected group. Cells were trypsinized 24 h post-infection, followed by fixation with 4% paraformaldehyde in 1× PBS for flow cytometry analysis. To avoid underestimation of the viral infectivity and cell loss due to the early detachment of infected cells from culture plate caused by Aβ_1-42_ treatments, infection rates of SARS-CoV-2 pseudovirus were adjusted to be under 20%. All cells suspended in the medium and on the plate were collected and were then subjected to flow cytometry analysis (BD FACScalibur, BD Biosciences, San Jose, CA, USA). The percentage of the cell count (5000 cells per sample) above the intensity of 100 at FL1-H is presented as infection rates of SARS-CoV-2 pseudovirus (*n* = 6 per group). For semi-quantification of the control pseudovirus infectivity, pseudovirus VSVΔG-G that did not express the S protein of SARS-CoV-2 was used. Infectivity was measured by total GFP fluorescent area per DAPI-positive cell observed in the pseudovirus VSVΔG-G-infected group (including 865 cells) and pseudovirus VSVΔG-G-infected plus Aβ_1-42_ groups at 1 (including 1036 cells), 10 (including 990 cells), or 50 µg/mL (including 924 cells). Randomly selected cells in two chambered-slides were used, and data are presented as ratios with respect to the pseudovirus VSVΔG-G-infected group.

### 4.4. IL-6 Expression in A549 Cells after Infection with SARS-CoV-2 Pseudovirus

A549 cells that were regularly maintained in F12K medium (Thermo Fisher, Waltham, MA, USA) containing 10% FBS, 1× P/S solution (Corning, Glendale, AZ, USA), 1× MEM-Eagle (Biological Industries, Cromwell, CT, USA), and 1× L-glutamine at 37 °C in a 5% CO_2_ incubator were used for cytokine expression. A549 cells were seeded at a density of 5 × 104 cells per well in a 4-well chambered slide overnight. Following the infection and treatments of Aβ_1-42_ at 50 µg/mL similar to the procedure described above, cells were fixed in 4% paraformaldehyde in 1× PBS 17 h after infection with the SARS-CoV-2 pseudovirus. To detect the intracellular IL-6 levels, immuno-fluorescence confocal microscopy using rabbit anti-human IL-6 antibody (Abcam, Cambridge, MA, USA) and Alexa Fluo 594-conjugated donkey anti-rabbit antibody was performed. Semi-quantification of IL-6 immuno-reactivity was measured by total fluorescent area of Alexa Fluo 594 per DAPI-positive cell for the control (including 1016 cells from 3 wells), Aβ_1-42_ alone at 50 µg/mL (including 778 cells from 2 wells), SARS-CoV-2 pseudovirus-infected group (868 cells from 3 wells), and SARS-CoV-2 pseudovirus infection in the presence of Aβ_1-42_ at 50 µg/mL (including 861 cells from 3 wells).

### 4.5. Functional ELISA Binding Assay

For examining Aβ_1-42_ binding to viral proteins, His-tagged viral proteins, including the S1 of SARS-CoV-2, HA of H1N1, and S1 of MERS-CoV purchased from Sino Biological (Wayne, PA, USA), were immobilized at 125 ng (100 µL) per well in a 96-well ELISA plate (Thermo Fisher Scientific, Waltham, MA, USA) at 4 °C for overnight incubation. Following blocking for 1.5 h in 1% BSA (1× PBS), serial dilution of Aβ_1-42_ was performed for 1 h, followed by washing and incubation with an anti-Aβ antibody, NP106, at 1:500 dilution (2 ng/mL) in 1× PBST (containing 1× PBS and 0.05% Tween 20) for 1 h. HRP-conjugated antibody against mouse IgG (Millipore Burlington, MA, USA) was used as a secondary antibody in 1× PBST (1:500 dilution, 2 ng/mL), followed by the addition of 3,3’5,5’-tetramethylbenzidine (TMB) substrate (Seracare Milford, MA, USA) for color development. The samples were then analyzed at 450 nm using a plate reader (SpectraMax M2, Molecular Devices San Jose, CA, USA). Binding assays for Aβ_1-42_ mutants (AnaSpec, Fremont, CA, USA) and Aβ_1-40_ (GeneZyme Biotechnology, Miaoli County, Taiwan) were similarly performed with immobilized S1 of SARS-CoV-2-His (125 ng/well), while immobilized RBD-His (provided by Antaimmu BioMed, Hsinchu, Taiwan) at 125 ng/well and hACE2-His (ACRO Biosystems, Newark, DE, USA) at 200 ng/well were also used for the binding with Aβ_1-42_. For the competitive binding assay, immobilized S1 of SARS-CoV-2-His (125 ng/well) was incubated with 1× PBS with Aβ_1-42_ at 50 or 200 ng per well for 1 h after blocking. This was followed by 1 h of incubation with serial dilutions of hACE2-Fc. HRP-conjugated anti-human IgG antibody (GeneTex, Irvine, CA, USA) and TMB substrate were used as described above.

### 4.6. Linear Binding Epitope of Viral Proteins on Aβ_1-42_

Biotin-conjugated Aβ_1-42_ fragments were custom-made by Mimotopes (Mimotopes Pty Ltd, Mulgrave, Victoria, Australia) as illustrated in Supplementary Figure S2, and each fragment contained 10 amino acids of Aβ_1-42_, with a difference of 2 amino acids for every two adjacent sequences. Experiments were performed per the manufacturer’s instructions. Briefly, streptavidin probes were coated in 96-well ELISA plates (Thermo Fisher Scientific, Waltham, MA, USA) overnight at 4 °C followed by blocking in 1% BSA for 1 h. After washing, each of the 17 peptide fragments of Aβ_1-42_ (1:100 dilution) was then incubated for 1 h. His-tagged viral proteins, including the S1 of SARS-CoV-2, HA of H1N1, and the S1 of MERS-CoV, and hACE2-Fc were applied at 100 ng per well (100 µL) for 1 h following washing. HRP-conjugated anti-His antibody (GeneTex, Irvine, CA, USA) and HRP-conjugated anti-human IgG antibody were used as described above, and samples were read at 450 nm with a SpectraMax M2 plate reader. Values of OD greater than 0.8 were considered to show an apparent binding ability to the fragments of Aβ_1-42_.

### 4.7. Interaction between hACE2-Fc and Aβ Plaques Examined by Confocal Microscopy

Following transcardial perfusion of PBS, the brain of APP/PS1 transgenic mice at the age of 16 months were collected and subjected to fixation with 4% paraformaldehyde in 1× PBS followed by 30% sucrose in 1× PBS for cryoprotection. Free-floating cryosections of 30 µm in thickness were treated with 88% formic acid for antigen retrieval followed by blocking with 1% BSA in 1× PBS for 1 h. Brain sections were then incubated with hACE2-Fc (10 µg/mL) overnight at 4 °C. For the blocking assay, Aβ_1-42_ (10 µg/mL) was pre-incubated with hACE2-Fc (10 µg/mL) at room temperature for 2 h before application onto the brain sections. After overnight incubation, brain sections were brought to room temperature and NP106, an N-terminal Aβ-specific mouse monoclonal antibody, was added at 2 µg/mL for 2 h for detection of Aβ plaques. After washing, Alexa Fluor 488-conjugated antibody against human IgG (1:200) was used to detect hACE2-Fc, and Alexa Fluor 594-conjugated antibody against mouse IgG (1:200) was used to detect Aβ plaques in the brain tissue. Mounting medium (Vector lab, Burlingame, CA, USA) containing 4,6-diamidino-2-phenylindole (DAPI) was used to label nuclei. Images were acquired using a Leica confocal microscopy imaging system.

### 4.8. Novel Aβ Antibody NP106: Linear Epitope Mapping and Binding Affinity to Aβ_1-42_

NP106 is a recombinant mouse monoclonal antibody specific for Aβ, which is derived from a mouse hybridoma monoclonal antibody generated by immunization of oligomeric Aβ_1-42_ in mice (LTK Biolaboratories, Taoyuan County, Taiwan). The cDNA encoding the antibody sequence was cloned into a pcDNA3.4 expression vector (Thermo Fisher Scientific, Waltham, MA, USA), and NP106 was expressed by an Expi-CHO cell system (Thermo Fisher Scientific, Waltham, MA, USA) according to the manufacturer’s protocol, followed by purification using protein G (GE Healthcare Chicago, IL, USA). To examine the binding affinity to Aβ, NP106 was applied to a 96-well ELISA plate coated with oligomeric Aβ_1-42_ (10 ng per well), followed by addition of HRP-conjugated anti-mouse antibody. TMB substrate was then used for color development, and samples were analyzed by using the SpectraMax M2 plate reader at 450 nm. As demonstrated in Supplementary Figure S1, NP106 binds strongly to various forms of Aβ_1-42_ (K_D_ < 5 nM), and the binding epitope is estimated to be located approximately at N-terminal residues 3 to 10 of Aβ_1-42_.

### 4.9. Measurement of Serum Aβ_1-42_ Levels in Mice with Intravenous Inoculation of Aβ_1-42_

The levels of serum Aβ_1-42_ were measured by using an ELISA kit (Invitrogen, Carlsbad, CA, USA) specific for Aβ_1-42_ per the manufacturer’s instructions. The absorbance was set at 450 nm using a SpectraMax M2 plate reader.

### 4.10. The Effects of SARS-CoV-2 SP_ECD_ Trimers on Aβ_1-42_ Clearance in the Blood

Production of SP_ECD_ trimers is briefly described as follows. His-tagged SP_ECD_ trimers were modified from Cryo-EM structures (PDBID:6VXX, http://dx.doi.org/10.1016/j.cell.2020.02.058 (accessed on 10 July 2020)). The trimerization element structure was adapted from the published X-ray structures (PDBID:4NCU, http://doi.org/10.2210/pdb4NCU/pdb (accessed on 10 July 2020)). His-tagged SP_ECD_ trimers were expressed in HEK293 cells followed by dialysis using buffer exchanging against 100x in a volume of His-Affinity chromatography Buffer A (containing 20 mM sodium phosphate, 0.5 M sodium chloride, 5 mM imidazole, pH 7.4) at 4 °C for 4 h. The dialyzed protein solution was clarified using a 0.2 μm filter before loading onto a pre-equilibrated (in His-AFC Buffer A) His-Trap HP column (GE Life Sciences 17-5248-01 or 17-5268-02). The bound His-tagged protein were eluted using a linear gradient (10~50% of 1 M imidazole) after two washing steps (5 CV of 4% and 10% imidazole) on an AKTA Pure 25 FPLC system. The fractions correlated to SP_ECD_ trimers were pooled, concentrated through a 30kDa MWCO ultrafiltration centrifugal filter, and diafiltrated for buffer exchanging into 1× PBS, pH 6.85. The molecular size of the native state of SP_ECD_ trimers was analyzed using size exclusion chromatography on a Superdex 200 Increase 10/300 GL column connected to an AKTA Pure 25 FPLC system. One hundred micrograms of the purified SP_ECD_ trimers was injected into the pre-equilibrated (in 1× PBS, pH 7.4) column at a flow rate of 0.5 mL/min. The elution was monitored by the absorbance at 280 nm in a 0.1 cm path-length flow cell. The relative molecular weight (MW) of the eluted peak was calculated from the following fitting equation: y = −0.2018x + 7.7007, (R2 = 0.9948), which was determined by plotting the elution volumes versus the log MW of the calibration standards in a High Molecular Weight Calibration Kit (GE Life Sciences, 28-4038-42). The SP_ECD_ trimers’ eluted peak (8.87 mL) correlated with the MW of 814 kDa. C57/B6 mice (at the age of approximately 4 months) were then injected intravenously with human Aβ_1-42_ (1 µg in 1× PBS) in the presence or the absence of 10 µg of SP_ECD_ trimers with or without NP106 (15 µg). Four groups, including Aβ_1-42_ alone (*n* = 5), Aβ_1-42_ + SP_ECD_ (*n* = 5), Aβ_1-42_ + SP_ECD_ + NP106 (*n* = 4), and Aβ_1-42_ + NP106 (*n* = 5), were used. Serum was collected at 15, 30, 60, and 120 min after injection followed by measuring Aβ_1-42_ levels using an ELISA kit (Thermo Fisher, Waltham, MA, USA) per the manufacturer’s instructions.

### 4.11. Statistical Analysis

A two-tailed independent Student’s *t*-test was used to test for statistical significance. For ANOVA, significance with post hoc multiple comparisons between groups was determined with the Bonferroni test using GraphPad Prism software. EC_50_ was estimated from the binding curve of mean values using GraphPad Prism software. Data are presented as mean ± SEM, unless specified otherwise. Statistical significance was set at *p* < 0.05.

## 5. Patents

NP106, formerly named mAβ10, is a novel anti-Aβ antibody protected by a United States patent (notice of allowance on 14 June 2021), Republic of China patent (#I721440 in 2021), and the Patent Cooperation Treaty (pending).

## Data Availability

Data of the current study are available from the corresponding author on request.

## Supplementary Materials

The following are available online at https://www.mdpi.com/article/10.3390/ijms22158226/s1.
