# Supplementary material for: The Effects of Aβ1-42 Binding to the SARS-CoV-2 Spike Protein S1 Subunit and Angiotensin-Converting Enzyme 2
Source: Int J Mol Sci. 2021 Jul 30;22(15):8226. doi: 10.3390/ijms22158226 (PMC8347908; doi:10.3390/ijms22158226)
Supplement: Supplementary file 1 [file ijms-22-08226-s001.zip › ijms-1305938-SI.pdf]

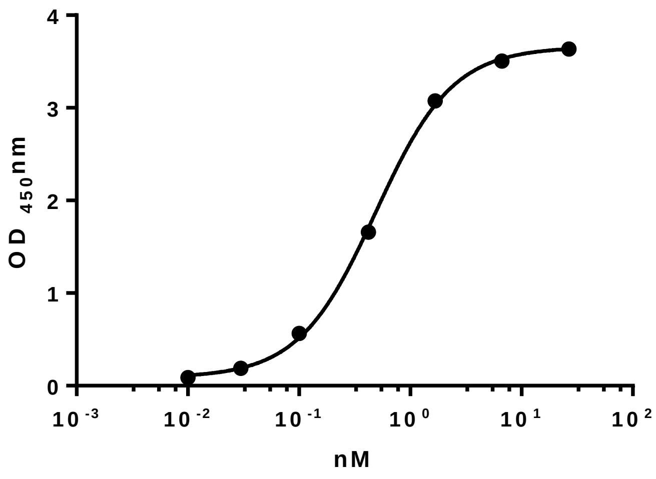

Figure S1. *Binding of NP106 to Aβ1-42.* As evaluated by ELISA, NP106 binds strongly to immobilized oligomeric $A\beta_{1\text{-}42}$ (oAβ).

$A\beta_{1-42}$: N- DAEFRHDSGYEVHHQKLVFFAEDVGSNKGAIIGLMVGGVVIA -C

Peptides:

1: biotin- DAEFRHDSGYGSG
2: biotin- SGSG EFRHDSGYEV
3: biotin- SGSG RHDSGYEVHH
4: biotin- SGSG DSGYEVHHQK
5: biotin- SGSG GYEVHHQKLV
6. biotin- SGSG EVHHQKLVFF
7. biotin- SGSG HHQKLVFFAE
8. biotin- SGSG QKLVFFAEDV
9. biotin- SGSG LVFFAEDVGS
10. biotin- SGSG FFAEDVGSNK
11. biotin- SGSG AEDVGSNKGA
12. biotin- SGSG DVGSNKGAIIG
13. biotin- SGSG GSNKGAIIGLM
14. biotin- SGSG NKGAIIGLMVG
15. biotin- SGSG GAIIGLMVGGV
16 biotin- SGSG IIGLMVGGVV
17. biotin- SGSG GLMVGGVVIA

Figure S2. *Design of 17-peptide fragments of* $A\beta_{1-42}$ *for linear epitope mapping of viral surface proteins*. Biotin-conjugated peptides containing 10 amino acids of $A\beta_{1-42}$ (in blue) were synthesised as indicated (every two adjacent sequences differ by two amino acids). Immobilized peptide fragments of $A\beta_{1-42}$ were used to predict the linear binding regions of viral surface proteins on $A\beta_{1-42}$.
